# Microbiota‐microglia connections in age‐related cognition decline

**DOI:** 10.1111/acel.13599

**Published:** 2022-03-29

**Authors:** Rui Zhou, Shufang Qian, William C. S. Cho, Jinyun Zhou, Chentao Jin, Yan Zhong, Jing Wang, Xiaohui Zhang, Zhoujiao Xu, Mei Tian, Lawrence W. C. Chan, Hong Zhang

**Affiliations:** ^1^ Department of Nuclear Medicine and Medical PET Center The Second Affiliated Hospital of Zhejiang University School of Medicine Hangzhou China; ^2^ 26680 Department of Health Technology and Informatics The Hong Kong Polytechnic University Hong Kong SAR China; ^3^ Department of Clinical Oncology Queen Elizabeth Hospital Hong Kong SAR China; ^4^ 12377 Key Laboratory for Biomedical Engineering of Ministry of Education Zhejiang University Hangzhou China; ^5^ The College of Biomedical Engineering and Instrument Science of Zhejiang University Hangzhou China

**Keywords:** cognitive aging, gut microbiota, microglia, neuroinflammation

## Abstract

Aging is an inevitable process that all individuals experience, of which the extent differs among individuals. It has been recognized as the risk factor of neurodegenerative diseases by affecting gut microbiota compositions, microglia, and cognition abilities. Aging‐induced changes in gut microbiota compositions have a critical role in orchestrating the morphology and functions of microglia through the gut‐brain axis. Gut microbiota communicates with microglia by its secreted metabolites and neurotransmitters. This is highly associated with age‐related cognitive declines. Here, we review the main composition of microbiota in the aged individuals, outline the changes of the brain in age‐related cognitive decline from a neuroinflammation perspective, especially the changes of morphology and functions of microglia, discuss the crosstalk between microbiota and microglia in the aged brain and further highlight the role of microbiota‐microglia connections in neurodegenerative diseases (Alzheimer's disease and Parkinson's disease).

## Graphical abstract

Microbiota‐microglia connection has been recognized in recent years. Aging‐induced changes of gut microbiota composition have a critical role in mediating the morphology and functions of microglia through gut‐brain axis, which is tightly related to age‐related cognitive decline. Here, we review the age‐related changes of gut microbiota and brain, and highlight the interaction between microbiota and microglia in the aged brain.

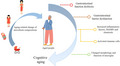

**Abbreviations**
Ach: acetylcholine
AD: Alzheimer’s disease
Aβ: amyloid‐beta
BBB: blood–brain barrier
BMDMs: bone marrow‐derived macrophages
CCL3: C‐C motif ligand 3
CNS: central nervous system
CSF1R: colony stimulating factor 1 receptor
DAMPs: danger‐associated molecular patterns
ERK: extracellular signal‐regulated kinase
FFAR2: SCFA receptor‐free fatty acid receptor 2
FTL: ferritin light chain
GABA: gamma‐aminobutyric acid
GI: gastrointestinal
HFD: high‐fat diet
HiPR‐FISH: high‐phylogenetic‐resolution microbiota mapping by fluorescence in situ hybridization
IL‐17A: interleukin‐17A
IL‐1R1: interleukin‐1 receptor 1
JNK: c‐Jun N‐terminal kinase
LPS: lipopolysaccharide
MAMPs: microbial‐associated molecular patterns
MCTs: monocarboxylate transporters
MDP: muramyl dipeptide
MHC‐II: major histocompatibility complex class II
NE: norepinephrine
NF‐kB: nuclear factor‐kappa B
NFTs: neurofibrillary tangles
NLR3: NLR family pyrin domain containing 3
PAMPs: pathogen‐associated molecular patterns
PD: Parkinson’s disease
PET: positron emission tomography
PRPs: pattern recognition receptors
ROS: reactive oxygen species
SCFAs: short‐chain fatty acids
Smad3: mothers against decapentaplegic homolog 3
SMCT: sodium‐dependent monocarboxylate transporter
ST‐seq: spatial transcriptomic sequencing
TGF‐β1: transforming growth factor β
TLRs: toll‐like receptors
Tregs: regulatory T cells

## 1 INTRODUCTION

Aging is defined as time‐dependent decrease of functions, which all the mammalian on earth will go through. Since the establishment of modern society, human beings, either from western or eastern societies, have struggled to discover a way to achieve longer life. In the past decades, the lifespan of citizens has been largely extended. Following this, the growing speed of the aging population is much faster than before, and the proportion of aged people over 60 years will increase to 22% at the end of 2050 (Organization, 2015).

Due to progressive loss of physiological function during aging, the human body will undergo a variety of biological and psychological changes, such as genomic instability, mitochondrial dysfunction, and athletic ability decline (Green et al., 2011; Moskalev et al., 2013; Stones, 2019). Brain, as the main part of the central nervous system (CNS), also suffers structural and molecular changes with increasing age (Fjell & Walhovd, 2010). The hallmarks of brain aging include impaired neuronal network activity, dysregulated glial cell activation, stem cell exhaustion, and dysregulated metabolic activity (Mattson & Arumugam, 2018). These accelerated alterations induce cognitive decline in old people, which might lead to neurodegenerative diseases, such as Parkinson's disease (PD) and Alzheimer's disease (AD) (Baldwin & Greenwood, 2020; Goldberg et al., 2018).

Microglia are a group of neuroglia that account for 5–15% of total brain cells (Pelvig et al., 2008). As the resident‐macrophage cells, microglia function as the main immune defense in the CNS. To sustain brain homeostasis, microglia continually surveille the brain microenvironment through their connections with neighboring cells and factors (D. Li et al., 2018). During aging, microglia switch from resting state to activated state and contribute to the development of neurogenerative diseases (Johnson et al., 2020). Activated microglia perform in an ameboid morphology, produce pro‐inflammatory cytokines, and participate in regulating blood–brain barrier (BBB) integrity and synaptic plasticity in aged brain (Daria et al., 2017; Phi T Nguyen et al., 2020; Yousef et al., 2019).

Recent studies suggested that the alterations of gut microbiota in the aged are associated with neurodegenerative diseases (Fujii et al., 2019). Gut‐brain axis indicates the complicated connections between gut and brain, which is crucial for microglial maturation and function (Erny et al., 2015; Fung et al., 2017). These findings pave a new way in attenuating and even reversing cognitive aging through microbiota‐microglia axis intervention. In this review, we will review the composition of gut microbiota in aged individuals, depict the changes of microglia associated with aging and discuss neuroinflammation in the aged brain. We then summarize the mechanism of microbiota in regulating microglial function in the aged brain and highlight the role of microbiota‐microglia connections in neurodegenerative diseases. This knowledge may enrich our understanding of the crosstalk between aging‐related cognitive decline and the microbiota‐microglia axis, facilitating the discovery of novel targets in restoring aging‐related cognitive decline.

## 2 THE COMPOSITION OF GUT MICROBIOTA IN THE AGED

### 2.1 Perturabation of Gut microbiota in the Adult

Currently, 2172 microorganism species, which are classified into 12 different phyla, have been identified in human beings (Hugon et al., 2015). Among them, 93.5% are occupied by *Proteobacteria*, *Firmicutes*, *Actinobacteria*, and *Bacteroidetes* (Thursby & Juge, 2017). In adults, gut microbiota communities remain relatively stable, while it could be disturbed by mental state, diet, pharmaceuticals, geography, and lifecycle stages.

The altered mental state is recognized as one of the main factors which are associated with gut dysbiosis. Recent evidence showed that anxiety is linked with unbalanced gut microorganisms and intestinal barrier disruption (Bruce R Stevens et al., 2018). Collected feces from human showed that *Acidaminococcaceae*, *Enterobacteriaceae*, *Rikenellaceae*, and *Coriobacteriaceae* families abundance are increased in patients with depression (Jiang et al., 2015; Stevens et al., 2020). Another systematic review showed that nine genera of bacteria were significantly abundant (*Anaerostipes*, *Blautia*, *Clostridium*, *Klebsiella*, *Lachnospiraceae incertae sedis*, *Parabacteroides*, *Parasutterella*, *Phascolarctobacterium*, *and Streptococcus*) in major depression disorders (Cheung et al., 2019). These species are mainly involved in butyrate and gamma‐aminobutyric acid (GABA) degradation pathways in depression (Valles‐Colomer et al., 2019). In the rodent model, chronic psychological stress induces translocation of *E. Coli* into enterocytes by more than 30 folds and enhanced permeability of HRP in enterocytes (Velin et al., 2004). Besides, psychological stress combined with filamentous bacteria inducing interleukin‐17A (IL‐17A) increases the gut permeability and promotes vaso‐occlusive episodes in patients with sickle cell disease (Xu et al., 2020). Antidepressant minocycline alleviates depressive behavior and increases abundance of *Lachnospiraceae* and *Clostridiales Family XIII* Families, which is known for butyrate production (Schmidtner et al., 2019). Moreover, gut microbiota dysbiosis‐induced damage of the intestinal barrier may make the gastrointestinal tract vulnerable to inflammation, which results in a feed‐forward cycle between gastrointestinal inflammation and barrier dysfunction and may induce the development of depression. A human study indicated that plasma level of lipopolysaccharide (LPS) endotoxin secreted by microbiota was elevated in patients with anxiety and depression and correlated with gastrointestinal barrier dysfunction, which might lead to anxiety and depression (Bruce R. Stevens et al., 2018).

The diet is another factor that alters the composition of gut microbiota. A high‐fat diet (HFD) is defined as a diet rich in fats, especially saturated fats, and is becoming a common lifestyle in human beings. Though numerous studies including rodent models and human have investigated the influence of HFD on microbiota communities, a meta‐analysis indicated that only the ratio of *Firmicutes* to *Bacteroidetes* was continuously increased in the 27 enrolled studies (Bisanz et al., 2019). Interestingly, a murine study showed that increased soluble fiber rather than fat in the diet significantly results in loss of the phylum *Bacteroidetes* and rise of *Clostridia* and *Proteobacteria* (Morrison et al., 2020).

### 2.2 Changed composition of gut microbiota in the elderly

The gastrointestinal tract in the elderly is more vulnerable to physiological changes and risk factors (Camilleri et al., 2000). Given the potential of gut microbiota to increase gastrointestinal permeability and influence gut inflammation through its secretion, considerable interest has appeared in the variation of gut microbiota composition at an advanced age. Generally, the diversity and stability of gut microbiota are declined during aging (Table 1), indicating gut dysbiosis in the elderly (Kong et al., 2019; Lynch & Pedersen, 2016). In the elderly, phylum *Bacteroidetes*, *Faecalibacterium* spp., and *Clostridium* IV are relatively abundant, while phylum *Firmicutes*, especially *Clostridium* XIVa, are more prevalent in the younger (Marcus J Claesson et al., 2011).

**TABLE 1:** Changed composition of gut microbiota in the elderly

Taxon (Phylum/Genus)		Trend	Country	Subjects amount	Composition of subjects	Method (Fecal Samples)	References
Unhealthy Aging							
Actinobacteria	Bifidobacterium	↓	Spain	153 subjects	49 adults (ages <50 years), 58 elderly (ages 50–65 years), 46 old (ages 66–80 (*n* = 19), and >80 (*n* = 27) years) subjects	qPCR	Salazar et al. (2019)

↓	Thailand	120 subjects	73 adults (ages 30–40 years) and 47 elderly (ages ≥65 years) subjects	16S rRNA sequencing	La‐Ongkham et al. (2020)
Bacteroidetes	Alistipes	↑	Ireland	170 subjects	161 elderly (ages ≥65 years), 9 control (ages 28–46 years) subjects	16S rRNA sequencing	M. J. Claesson et al. (2011)

Bacteroides	↑	Finland	32 subjects	9 elderly non‐steroidal anti‐inflammatory drugs (NSAID) users (ages 77–85 years), 9 elderly non‐users (ages 70–83 years), and 14 young adults (ages 21–39 years)	%G + C profiling/16S rDNA sequencing	Mäkivuokko et al. (2010)

	↑	Ireland	170 subjects	161 elderly (ages ≥65 years), 9 control (ages 28–46 years) subjects	16S rRNA sequencing	M. J. Claesson et al. (2011)

	↑	Thailand	120 subjects	73 adults (ages 30–40 years) and 47 elderly (ages ≥65 years) subjects	16S rRNA sequencing	La‐Ongkham et al. (2020)

↓	India	54 subjects	12 young (ages 3–15 years), 18 elderly (ages 25–40 years) and 24 old (ages >50 years)	16S rRNA sequencing	Chaudhari et al. (2020)

↓	Spain	76 subjects	38 old subjects in retirement homes (ages 77–95 years), 38 elderly subjects in their own homes (ages 57–67 years)	qPCR	Salazar et al. (2013)

↓	Spain	153 subjects	49 adults (ages <50 years), 58 elderly (ages 50–65 years), 46 old (ages 66–80 (*n* = 19), and >80 (*n* = 27) years) subjects	qPCR	Salazar et al. (2019)

Parabacteroides	↑	Thailand	120 subjects	73 adults (ages 30–40 years) and 47 elderly (ages ≥65 years) subjects	16S rRNA sequencing	La‐Ongkham et al. (2020)
Firmicutes/Bacteroidetes		↓	Thailand	120 subjects	73 adults (ages 30–40 years) and 47 elderly (ages ≥65 years) subjects	16S rRNA sequencing	La‐Ongkham et al. (2020)
Firmicutes	Clostridium	↑	Finland	32 subjects	9 elderly NSAID users (ages 77–85 years), 9 elderly non‐users (ages 70–83 years), and 14 young adults (ages 21–39 years)	%G + C profiling/16S rDNA sequencing	Mäkivuokko et al. (2010)

↓	Spain	76 subjects	38 old subjects in retirement homes (ages 77–95 years), 38 elderly subjects in their own homes (ages 57–67 years)	qPCR	Salazar et al. (2013)

Coprobacillus	↓	Finland	32 subjects	9 elderly NSAID users (ages 77–85 years), 9 elderly non‐users (ages 70–83 years), and 14 young adults (ages 21–39 years)	%G + C profiling/16S rDNA sequencing	Mäkivuokko et al. (2010)

Dialister	↓	Finland	32 subjects	9 elderly NSAID users (ages 77–85 years), 9 elderly non‐users (ages 70–83 years), and 14 young adults (ages 21–39 years)	%G + C profiling/16S rDNA sequencing	Mäkivuokko et al. (2010)

Dorea	↓	Thailand	120 subjects	73 adults (ages 30–40 years) and 47 elderly (ages ≥65 years) subjects	16S rRNA sequencing	La‐Ongkham et al. (2020)

Faecalibacterium	↑	Ireland	170 subjects	161 elderly (ages ≥65 years), 9 control (ages 28–46 years) subjects	16S rRNA sequencing	M. J. Claesson et al. (2011)

	↓	Spain	153 subjects	49 adults (ages <50 years), 58 elderly (ages 50–65 years), 46 old (ages 66–80 (*n* = 19), and >80 (*n* = 27) years) subjects	qPCR	Salazar et al. (2019)

↓	Spain	76 subjects	38 old subjects in retirement homes (ages 77–95 years), 38 elderly subjects in their own homes (ages 57–67 years)	qPCR	Salazar et al. (2013)

Lactobacillus	↑	Finland	32 subjects	9 elderly NSAID users (ages 77–85 years), 9 elderly non‐users (ages 70–83 years), and 14 young adults (ages 21–39 years)	%G + C profiling/16S rDNA sequencing	Mäkivuokko et al. (2010)

↑	Spain	76 subjects	38 old subjects in retirement homes (ages 77–95 years), 38 elderly subjects in their own homes (ages 57–67 years)	qPCR	Salazar et al. (2013)

Lachnospiraceae	↓	Ireland	170 subjects	161 elderly (ages ≥65 years), 9 control (ages 28–46 years) subjects	16S rRNA sequencing	M. J. Claesson et al. (2011)

Oscillospira	↑	Finland	32 subjects	9 elderly NSAID users (ages 77–85 years), 9 elderly non‐users (ages 70–83 years), and 14 young adults (ages 21–39 years)	%G + C profiling/16S rDNA sequencing	Mäkivuokko et al. (2010)

Roseburia	↓	Finland	32 subjects	9 elderly NSAID users (ages 77–85 years), 9 elderly non‐users (ages 70–83 years), and 14 young adults (ages 21–39 years)	%G + C profiling/16S rDNA sequencing	Mäkivuokko et al. (2010)

Ruminococcus	↓	Finland	32 subjects	9 elderly NSAID users (ages 77–85 years), 9 elderly non‐users (ages 70–83 years), and 14 young adults (ages 21–39 years)	%G + C profiling/16S rDNA sequencing	Mäkivuokko et al. (2010)

Sporobacter	↑	Finland	32 subjects	9 elderly NSAID users (ages 77–85 years), 9 elderly non‐users (ages 70–83 years), and 14 young adults (ages 21–39 years)	%G + C profiling/16S rDNA sequencing	Mäkivuokko et al. (2010)

Streptococcus	↑	Finland	32 subjects	9 elderly NSAID users (ages 77–85 years), 9 elderly non‐users (ages 70–83 years), and 14 young adults (ages 21–39 years)	%G + C profiling/16S rDNA sequencing	Mäkivuokko et al. (2010)
Verrucomicrobia	Akkermansia	↑	Spain	76 subjects	38 old subjects in retirement homes (ages 77–95 years), 38 elderly subjects in their own homes (ages 57–67 years)	qPCR	Salazar et al. (2013)

The composition of gut microbiota in the elderly is disturbed by several factors, such as residence situation, exercise, nutrition, and age‐associated alterations. The residence location of the elderly may affect the composition of gut microbiota. The elderly who received long‐term residential care showed an increased proportion of phylum *Bacteroidetes*, genera *Parabacteroides*, *Eubacterium*, *Anaerotruncus*, *Lactonifactor*, and *Coprobacillus* compared with the community‐dwelling elderly (Claesson et al., 2012). Meanwhile, the movement was associated with the changed composition of the elderly with a reduced proportion of *Ruminococcus* and *Prevotella* and increased *Oscillibacter* (Claesson et al., 2012). Age‐induced deterioration in anatomical barriers and physiological functions also involves in the composition adjustment of gut microbiota in the elderly. In older individuals, elevated pro‐inflammatory factors, such as IL‐6, have a direct impact on intestinal permeability accompanied with decreased anti‐inflammatory factors (Nicoletti, 2015). The impaired intestinal barrier leads to an increased proportion of LPS‐secreting bacteria, such as *Bacteroides* and *Proteobacteria* (Kumar et al., 2016; Schiffrin et al., 2010). Apart from anatomical changes in the gastrointestinal (GI) tract during aging, deterioration in digestive function, such as occlusal force, muscle thickness, swallowing function, and secretive function, could lead to less fiber‐containing foods, which is a risk of malnutrition (Amarya et al., 2015). Malnutrition is reported to be related to an abundant subpopulation of *Clostridiales* (Jeffery et al., 2016). Immunity, as an important force in defending pathogens, has been shown a role in regulating gut microbiota (Vijay‐Kumar et al., 2010). Zhang et al. revealed that lack of adaptive immune system in mice resulted in elevated diversity and evenness of gut microbiota with an increase in age, which performed as an increased abundance of *A*. *muciniphila* colonization (H. Zhang et al., 2015). More evidence is required to reveal the role of the immune system in regulating gut microbiota during aging.

Centenarians are a group of people who have an extended lifespan than the normal elderly. The development of the next‐generation sequencing approach sheds a light on describing the composition of gut microbiota in the centenarians. Recently, numerous studies indicated that the composition of centenarians is different from the elderly and younger adults (Table 2). In a study from Italy, analysis of 21 centenarians revealed increased abundance of *Bacilli*, *Bacteroidetes*, and *Proteobacteria* compared with the elderly (Biagi et al., 2010). Meanwhile, microbiota sequencing of different ages (young adults, younger elderly, centenarians, and semisupercentenarians) indicated a decreased trend in the abundance of *Coprococcus*, *Roseburia*, and *Faecalibacterium* (E. Biagi et al., 2016). It is worth noting that the abundance of *Akkermansia* and *Escherichia* was significantly increased in the centenarians (E. Biagi et al., 2016). *Akkermansia*, known as the short‐chain fatty acids (SCFAs) producers, plays a critical role in anti‐inflammatory (Elena Biagi et al., 2016; Fanli Kong et al., 2016). This result was also confirmed by studies from China and Korea, which indicated the important role of these bacteria in the centenarians (B. S. Kim et al., 2019; F. Kong et al., 2016). An increased proportion of specific group of gut microbiota in centenarians might be associated with anti‐inflammatory effects and extended lifespan.

**TABLE 2:** Unique composition of gut microbiota in centenarians

Taxon (Phylum/Genus)		Trend	Country	Subjects amount	Subjects composition	Method (Fecal Samples)	References
Healthy Aging							
Actinobacteria	Bifidobacterium	↓	Italy	9 samples	3 centenarians (ages 99–102 years), 5 elderly (ages 59–75 years) and 1 adult (age 38 years)	Shotgun metagenomic sequencing	Rampelli et al. (2013)
		↑	Italy	65 subjects	21 old (ages 99–107 years), 25 elderly (ages 68–88 years), 19 young (ages 21–33 years) subjects	DNA library construction/Shotgun metagenomic sequencing	Wu et al. (2019)
		↑	Italy	39 subjects, 39 samples	24 semisupercentenarians (ages 105–109 years), 15 centenarians (ages 99–104 years), 15 elderly (ages 65–75 years), 15 young (ages 22–48 years)	16S rRNA sequencing	E. Biagi et al. (2016)
		↓	China	1195 subjects	86 elderly, 198 centenarians	16S rRNA sequencing	Bian et al. (2017)
		↓	Italy	84 subjects	21 centenarians (ages 99–104 years), 22 elderly (ages 63–76 years), 20 young (ages 25–40 years), 21 elderly (59–78 years, offspring of the centenarians) subjects	HITChip and quantitative PCR of 16S rRNA genes	Biagi et al. (2010)
		↓	Japan	367 subjects	ages between 0–104 years	16S rRNA sequencing	Odamaki et al. (2016)
	Collinsella	↑	Korea	56 subjects	30 centenarians (ages 95–108 years), 17 elderly subjects (ages 67–79 years), 9 adults (ages 26–43 years) in longevity villages	16S rRNA sequencing	B. S. Kim et al. (2019)
	Eggerthella	↑	Italy	84 subjects	21 centenarians (ages 99–104 years), 22 elderly (ages 63–76 years), 20 young (ages 25–40 years), 21 elderly (59–78 years, offspring of the centenarians) subjects	HITChip and quantitative PCR of 16S rRNA genes	Biagi et al. (2010)
		↑	Italy	62 samples	11 young (ages 22–48 years), 13 elderly (ages 65–75 years), 15 centenarians (ages 99–104 years), and 23 semisupercentenarians (ages 105–109 years) subjects	Shotgun metagenomic sequencing	Rampelli et al. (2020)
Bacteroidetes	Bacteroides	↑	Japan	367 subjects	ages between 0 and 104 years	16S rRNA sequencing	Odamaki et al. (2016)

↑		China	1195 subjects	86 elderly, 198 centenarians	16S rRNA sequencing	Bian et al. (2017)

↓	Italy	62 samples	11 young (ages 22–48 years), 13 elderly (ages 65–75 years), 15 centenarians (ages 99–104 years), and 23 semisupercentenarians (ages 105–109 years) subjects	Shotgun metagenomic sequencing	Rampelli et al. (2020)

Parabacteroides	↑	Italy	65 subjects	19 young (ages 19–33 years), 24 elderly (ages 68–88 years), 22 centenarians (ages >100 years) subjects	16S rRNA sequencing/ITS1 sequencing	Wu et al. (2020)

Prevotella	↓	Italy	65 subjects	19 young (ages 19–33 years), 24 elderly (ages 68–88 years), 22 centenarians (ages >100 years) subjects	16S rRNA sequencing/ITS1 sequencing	Wu et al. (2020)

	↓	Italy	65 subjects	21 old (ages 99–107 years), 25 elderly (ages 68–88 years), 19 young (ages 21–33 years) subjects	DNA library construction/shotgun metagenomic sequencing	Wu et al. (2019)

↑	China	1195 subjects	86 elderly, 198 centenarians	16S rRNA sequencing	Bian et al. (2017)

↓	Korea	56 subjects	30 centenarians (ages 95–108 years), 17 elderly subjects (ages 67–79 years), 9 adults (ages 26–43 years) in longevity villages	16S rRNA sequencing	B. S. Kim et al. (2019)
Euryarchaeota	Methanobrevibacter	↑	Italy	65 subjects	21 old (ages 99–107 years), 25 elderly (ages 68–88 years), 19 young (ages 21–33 years) subjects	DNA library construction/shotgun metagenomic sequencing	Wu et al. (2019)
		↑	Italy	62 samples	11 young (ages 22–48 years), 13 elderly (ages 65–75 years), 15 centenarians (ages 99–104 years), and 23 semisupercentenarians (ages 105–109 years) subjects	Shotgun metagenomic sequencing	Rampelli et al. (2020)
Firmicutes	Bacillus	↑	Italy	84 subjects	21 centenarians (ages 99–104 years), 22 elderly (ages 63–76 years), 20 young (ages 25–40 years), 21 elderly (59–78 years, offspring of the centenarians) subjects	HITChip and quantitative PCR of 1S rRNA genes	Biagi et al. (2010)

Clostridium	↑	China	1195 subjects	86 elderly, 198 centenarians	16S rRNA sequencing	Bian et al. (2017)

	↑	Korea	56 subjects	30 centenarians (ages 95–108 years), 17 elderly subjects (ages 67–79 years), 9 adults (ages 26–43 years) in longevity villages	16S rRNA sequencing	B. S. Kim et al. (2019)

↓	Italy	84 subjects	21 centenarians (ages 99–104 years), 22 elderly (ages 63–76 years), 20 young (ages 25–40 years), 21 elderly (59–78 years, offspring of the centenarians) subjects	HITChip and quantitative PCR of 16S rRNA genes	Biagi et al. (2010)

Coprococcus	↓	Italy	65 subjects	21 old (ages 99–107 years), 25 elderly (ages 68–88 years), 19 young (ages 21–33 years) subjects	DNA library construction/shotgun metagenomic sequencing	Wu et al. (2019)

↓	Italy	62 samples	11 young (ages 22–48 years), 13 elderly (ages 65–75 years), 15 centenarians (ages 99–104 years), and 23 semisupercentenarians (ages 105–109 years) subjects	Shotgun metagenomic sequencing	Rampelli et al. (2020)

Dorea	↑	China	1195 subjects	86 elderly, 198 centenarians	16S rRNA sequencing	Bian et al. (2017)

↓	Italy	65 subjects	21 old (ages 99–107 years), 25 elderly (ages 68–88 years), 19 young (ages 21–33 years) subjects	DNA library construction/shotgun metagenomic sequencing	Wu et al. (2019)

Roseburia	↓	Italy	84 subjects	21 centenarians (ages 99–104 years), 22 elderly (ages 63–76 years), 20 young (ages 25–40 years), 21 elderly (59–78 years, offspring of the centenarians) subjects	HITChip and quantitative PCR of 16S rRNA genes	Biagi et al. (2010)

Ruminococcus	↓	Italy	65 subjects	19 young (ages 19–33 years), 24 elderly (ages 68–88 years), 22 centenarians (ages >100 years) subjects	16S rRNA sequencing/ITS1 sequencing	Wu et al. (2020)


↑	Italy	9 samples	3 centenarians (ages 99–102 years), 5 elderly (ages 59–75 years) and 1 adult (age 38 years)	Shotgun metagenomic sequencing	Rampelli et al. (2013)


↓	Italy	84 subjects	21 centenarians (ages 99–104 years), 22 elderly (ages 63–76 years), 20 young (ages 25–40 years), 21 elderly (59–78 years, offspring of the centenarians) subjects	HITChip and quantitative PCR of 16S rRNA genes	Biagi et al. (2010)


↓	Italy	65 subjects	21 old (ages 99–107 years), 25 elderly (ages 68–88 years), 19 young (ages 21–33 years) subjects	DNA library construction/shotgun metagenomic sequencing	Wu et al. (2019)

Eubacterium	↓	Italy	9 samples	3 centenarians (ages 99–102 years), 5 elderly (ages 59–75 years), and 1 adult (age 38 years)	Shotgun metagenomic sequencing	Rampelli et al. (2013)

	↓	Italy	84 subjects	21 centenarians (ages 99–104 years), 22 elderly (ages 63–76 years), 20 young (ages 25–40 years), 21 elderly (59–78 years, offspring of the centenarians) subjects	HITChip and quantitative PCR of 16S rRNA genes	Biagi et al. (2010)

↓	Italy	65 subjects	21 old (ages 99–107 years), 25 elderly (ages 68–88 years), 19 young (ages 21–33 years) subjects	DNA library construction/shotgun metagenomic sequencing	Wu et al. (2019)

↓	Italy	62 samples	11 young (ages 22–48 years), 13 elderly (ages 65–75 years), 15 centenarians (ages 99–104 years), and 23 semisupercentenarians (ages 105–109 years) subjects	Shotgun metagenomic sequencing	Rampelli et al. (2020)

Faecalibacterium	↓	Italy	9 samples	3 centenarians (ages 99–102 years), 5 elderly (ages 59–75 years), and 1 adult (age 38 years)	Shotgun metagenomic sequencing	Rampelli et al. (2013)

	↑	China	1195 subjects	86 elderly, 198 centenarians	16S rRNA sequencing	Bian et al. (2017)

	↓	China	67 subjects	centenarians and nonagenarians (ages ≥90 years)	16S rRNA sequencing	Kong et al. (2019)

	↓	Italy	84 subjects	21 centenarians (ages 99–104 years), 22 elderly (ages 63–76 years), 20 young (ages 25–40 years), 21 elderly (59–78 years, offspring of the centenarians) subjects	HITChip and quantitative PCR of 16S rRNA genes	Biagi et al. (2010)

↓	Italy	65 subjects	21 old (ages 99–107 years), 25 elderly (ages 68–88 years), 19 young (ages 21–33 years) subjects	DNA library construction/shotgun metagenomic sequencing	Wu et al. (2019)

↓	Italy	62 samples	11 young (ages 22–48 years), 13 elderly (ages 65–75 years), 15 centenarians (ages 99–104 years), and 23 semisupercentenarians (ages 105–109 years) subjects	Shotgun metagenomic sequencing	Rampelli et al. (2020)

↓	Korea	56 subjects	30 centenarians (ages 95–108 years), 17 elderly subjects (ages 67–79 years), 9 adults (ages 26–43 years) in longevity villages	16S rRNA sequencing	B. S. Kim et al. (2019)

Lachnospiraceae	↓	Japan	367 subjects	ages between 0 and 104 years	16S rRNA sequencing	Odamaki et al. (2016)

Lactobacillus	↑	Italy	65 subjects	19 young (ages 19–33 years), 24 elderly (ages 68–88 years), 22 centenarians (ages >100 years) subjects	16S rRNA sequencing	Wu et al. (2020)


↑	Spain	153 subjects	49 adults (ages <50 years), 58 elderly (ages 50–65 years), 46 old (ages 66–80 (*n* = 19), and >80 (*n* = 27) years) subjects	qPCR	Salazar et al. (2019)

Marvinbryantia	↑	China	1195 subjects	86 elderly, 198 centenarians	16S rRNA sequencing	Bian et al. (2017)
Proteobacteria	Desulfovibrio	↑	Italy	65 subjects	21 old (ages 99–107 years), 25 elderly (ages 68–88 years), 19 young (ages 21–33 years) subjects	DNA library construction/shotgun metagenomic sequencing	Wu et al. (2019)
	Escherichia/Shigella	↑	China	67 subjects	centenarians and nonagenarians (ages ≥90 years)	16S rRNA sequencing	Kong et al. (2019)
	Escherichia	↑	Italy	9 samples	3 centenarians (ages 99–102 years), 5 elderly (ages 59–75 years) and 1 adult (age 38 years)	Shotgun metagenomic sequencing	Rampelli et al. (2013)
		↑	Italy	62 samples	11 young (ages 22–48 years), 13 elderly (ages 65–75 years), 15 centenarians (ages 99–104 years), and 23 semisupercentenarians (ages 105–109 years) subjects	Shotgun metagenomic sequencing	Rampelli et al. (2020)
		↑	Korea	56 subjects	30 centenarians (ages 95–108 years), 17 elderly subjects (ages 67–79 years), 9 adults (ages 26–43 years) in longevity villages	16S rRNA sequencing	B. S. Kim et al. (2019)
	Klebsiella	↑	Italy	84 subjects	21 centenarians (ages 99–104 years), 22 elderly (ages 63–76 years), 20 young (ages 25–40 years), 21 elderly (59–78 years, offspring of the centenarians) subjects	HITChip and quantitative PCR of 16S rRNA genes	Biagi et al. (2010)
	Vibrio	↑	Italy	84 subjects	21 centenarians (ages 99–104 years), 22 elderly (ages 63–76 years), 20 young (ages 25–40 years), 21 elderly (59–78 years, offspring of the centenarians) subjects	HITChip and quantitative PCR of 16S rRNA genes	Biagi et al. (2010)
Verrucomicrobia	Akkermansia	↑	Italy	84 subjects	21 centenarians (ages 99–104 years), 22 elderly (ages 63–76 years), 20 young (ages 25–40 years), 21 elderly (59–78 years, offspring of the centenarians) subjects	HITChip and quantitative PCR of 16S rRNA genes	Biagi et al. (2010)
		↑	Italy	39 subjects, 39 samples	24 semisupercentenarians (ages 105–109 years), 15 centenarians (ages 99–104 years), 15 elderly (ages 65–75 years), 15 young (ages 22–48 years)	16S rRNA sequencing	E. Biagi et al. (2016)
		↑	Italy	62 samples	11 young (ages 22–48 years), 13 elderly (ages 65–75 years), 15 centenarians (ages 99–104 years), and 23 semisupercentenarians (ages 105–109 years) subjects	Shotgun metagenomic sequencing	Rampelli et al. (2020)
		↑	China	168 subjects	67 centenarians and nonagenarians, 54 elderly, and 47 young adults	16S rRNA sequencing	F. Kong et al. (2016)
		↑	Korea	56 subjects	30 centenarians (ages 95–108 years), 17 elderly subjects (ages 67–79 years), 9 adults (ages 26–43 years) in longevity villages	16S rRNA sequencing	B. S. Kim et al. (2019)
		↑	Spain	153 subjects	49 adults (ages <50 years), 58 elderly (ages 50–65 years), 46 old (ages 66–80 (*n* = 19), and >80 (*n* = 27) years) subjects	qPCR	Salazar et al. (2019)
Synergistetes	Cloacibacillus	↑	Italy	65 subjects	19 young (ages 19–33 years), 24 elderly (ages 68–88 years), 22 centenarians (ages >100 years) subjects	16S rRNA sequencing/ITS1 sequencing	Wu et al. (2020)
	Pyramidobacter	↑	Italy	65 subjects	21 old (ages 99–107 years), 25 elderly (ages 68–88 years), 19 young (ages 21–33 years) subjects	DNA library construction/shotgun metagenomic sequencing	Wu et al. (2019)

## 3 ALTERATIONS IN MICROGLIA DURING AGE‐RELATED COGNITIVE DECLINE

Cognitive aging is an inevitable and natural process due to the physiological decline during aging (Harada et al., 2013). It should be distinguished from mild cognitive impairment, dementia, and other specific cognitive decline syndromes. However, not all mental abilities are declined during aging. Verbal ability, some numerical abilities, and semantic knowledge are stable until the late life (Hedden & Gabrieli, 2004). Conversely, mental functions related to memory, such as working memory, episodic memory, and problem‐solving, have a tendency to decline in the aged (Craik, 1994). Alterations in morphology and functions of microglia and followed neuroinflammation in the brain could lead to age‐related cognitive decline.

### 3.1 Age‐related changes in morphology of microglia

Microglia, a group of resident immune cells in the brain, play a critical role in mediating neurogenesis, remodeling synapses, and regulating neuroinflammatory (Phi T. Nguyen et al., 2020; Pelvig et al., 2008; Wolfgang J. Streit et al., 2004). Recently, increasing studies indicated that microglia participated in several neurodegenerative diseases (V. Hugh Perry et al., 2010). At the stage of adult, microglia remain relatively homeostasis and the state of microglia is frequently mediated by factors existed in microenvironment of CNS (Van Rossum & Hanisch, 2004).

During the old age, obviously, morphological changes could be observed in microglia. Dystrophic microglia are a common group of microglia abundantly identified in aged human beings. A study including two nondemented individuals (38 and 68 years old) showed that dystrophic microglia presented with decreased fine branches, shortened tortuous processes, and/or formation of spheroids accompanied with significantly increased amount of dystrophic microglia in the aged brain (Wolfgang J. Streit et al., 2004). Similarly, Davies et al. observed reduced arborized area and length of processes in microglia during aging (Davies et al., 2017). With the progress of senescence, microglia show discontinuous processes and completely separate fragments of microglial cytoplasm under Iba‐1 staining in the AD cases (Davies et al., 2017; Tischer et al., 2016). Dystrophic microglia are also found to be close to Aβ plaques in postmortem AD patients (W. J. Streit et al., 2009). The markedly changed morphology of dystrophic microglia in the aged brain and AD supports the hypothesis that microglia experience a process of senescence during the course of aging, losing their abilities to respond to chronic inflammatory stimuli and no longer performing their neuroprotective duties (Bachstetter et al., 2015). Hypertrophic microglia or primed microglia are used to describe activated microglia in the brain, which could be observed in AD and accelerated aging (D. D. Raj et al., 2014; Walker & Lue, 2015). This type of microglia shows increased response to immune stimuli resulting in exaggerated inflammatory responses (V. H. Perry & Holmes, 2014). The morphological changes of hypertrophic microglia are different from that of dystrophic microglia. In an accelerated aging model of mice, microglia existed enlarged cell soma and thickened length of processes (D. D. Raj et al., 2014). No specific molecular markers are used to identify hypertrophic or primed microglia, while they express markers of activated macrophages, such as MHC‐II, CD11b, and IL‐1β, when stimulated by pathogens and high‐fat diet (Butler et al., 2020; Garner et al., 2018). The distribution of hypertrophic microglia is inhomogeneous in the aged brain. Hypertrophic microglia are frequently distributed in atrophied gray matter and left language regions compared with resting microglia (Ohm et al., 2021). Meanwhile, in the rhesus monkey, increased density of hypertrophic microglia in frontal white matter was associated with cognitive impairment (Shobin et al., 2017). It is shown that hypertrophic microglia were extremely close to amyloid‐beta (Aβ) plaques in several brain areas, especially cortex and hippocampus in aged human cases and transgenic mouse model of AD (Bouvier et al., 2016). In this situation, hypertrophic microglia showed upregulated CD33 expression, which is associated with decreased clearance and accumulation of Aβ plaques (Griciuc et al., 2013).

### 3.2 Age‐related changes in functions of microglia

Apart from the morphological change, the function of microglia is also changed during the process of priming. Accumulated pathogen‐associated molecular patterns (PAMPs) or danger‐associated molecular patterns (DAMPs), such as Aβ, tau, and α‐synuclein, in the aged brain induced microglial activation (Cai et al., 2014). Phagocytosis is an important function of microglia to clear dead cells, debris, and pathogens in the brain. Phagocytic receptors including CD36, TREM2b, and CD14 were found to upregulate in primed microglia (Raj et al., 2014). These microglia migrate to amyloid plaques and neurofibrillary tangles (NFTs) and attempt to sweep away them. However, in AD patients, microglia failed to effectively uptake amyloid plaque to prevent AD progression (Bamberger et al., 2003). Microglia adjacent to amyloid plaque showed impaired Ca^2+^ signaling characterized as increased frequency of spontaneous intracellular Ca^2+^ transients accompanied with smaller size (Brawek et al., 2014). Increased Ca^2+^ transients in vicinity of plaque may induce the release of pro‐inflammatory cytokines, such as TNF‐α and NO, which is relied on the concentration of Ca^2+^, deteriorate the neuroinflammation, and promote AD progression (Färber & Kettenmann, 2006; Hide et al., 2000). Toll‐like receptors (TLRs) are a group of family in pattern recognition, which is the crucial receptors in mediating phagocytosis of microglia. Upregulated TLR4 expression was observed in brain areas of AD patients, such as entorhinal cortex (Walter et al., 2007). On the one hand, TLR4‐mediated microglia activation, such as LPS (a classical TLR4 ligand) stimulation, induced clearance of Aβ plaques by microglial phagocytosis (Tahara et al., 2006). On the other hand, microglia activation mediated by TLR4 triggers the release of pro‐inflammatory cytokines (Papageorgiou et al., 2016). Besides, NLR family pyrin domain containing 3 (NLR3) inflammasome is aberrantly stimulated by PAMPs/DAMPs, which switches microglia into a pro‐inflammatory state (Ślusarczyk et al., 2018). Microglia also show increased proliferation as well as enhanced and uncontrolled inflammatory response to peripheral inflammatory factors, such as LPS, in a mouse model featured with accelerating aging (Raj et al., 2014). Single‐cell transcriptomic approaches indicated that iron metabolism‐related proteins, such as ferritin light chain (FTL), were differentially activated in dystrophic microglia in the aged brain (Shahidehpour et al., 2021). In a bioinformatic study based on HuMi_Aged gene, the proteome of aged human microglia showed that some signaling pathways, such as the ER‐phagosome pathway, endosomal pathway, and antigen processing‐cross presentation, were upregulated in aged human microglia (Olah et al., 2018). In general, morphological and functional changes of microglia during aging are likely to participate in the progression of cognitive aging.

### 3.3 Neuroinflammation in cognitive aging

**FIGURE 1:**
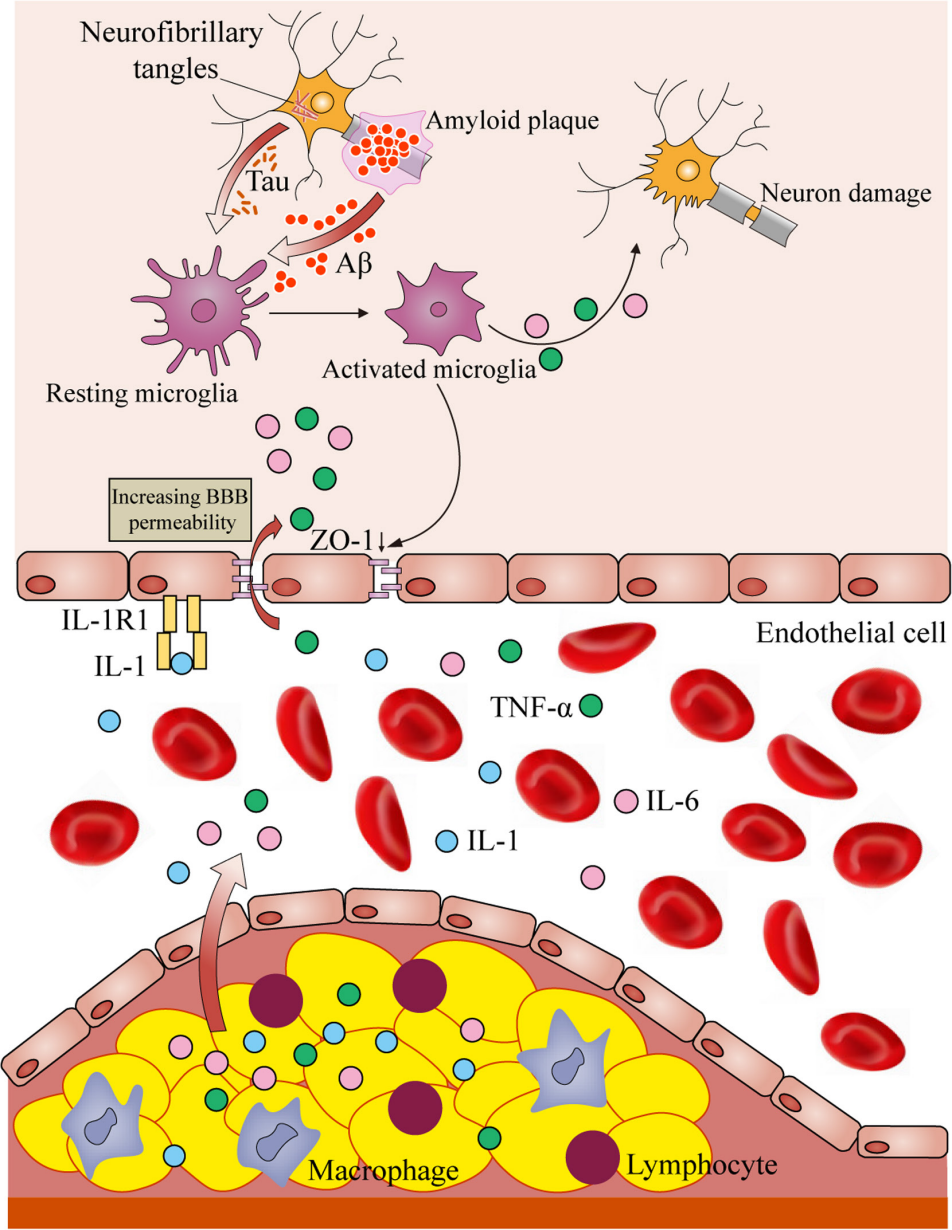
Inflammatory factors from atherosclerosis and microglia promoting neuroinflammation. Atheromatous plaques induced macrophages and lymphocytes infiltration in blood vessels released TNF‐α, IL‐6, and IL‐1 into blood circulation. These inflammatory factors across the leaky BBB in the aged brain and activate the microglia. Constantly existed Aβ and tau stimulate microglia and switch them from resting state into activated state. Activated microglia secreted IL‐6 and TNF‐α induce neuroinflammation and neuronal damage as well as BBB disruption

Neuroinflammation is defined as the inflammation in the nervous tissue. Emerging evidence shows that neuroinflammation plays a vital role in the cognitive decline of older population. Normally, the CNS is isolated from inflammatory factors or pathogens by BBB, a specifically selective semipermeable structure composed of pericytes, endotheliocytes, and astrocytes. However, BBB leakage is observed in aged people, and 13.5% of the old population is found to have BBB dysfunction (Bowman et al., 2018). It is hard to define whether BBB breakdown happens before neuroinflammation in the aged. Factors from outside (periphery) and inside (CNS) might both be involved in BBB breakdown and neuroinflammation development (Figure 1).

Microbiota also involve in mediating the function of BBB, though the mechanism underlying microbiota‐BBB connections is still foggy. A previous study observed increased BBB leakage in germ‐free mice combined with decreased occludin and claudin‐5 expression, which indicated the role of gut microbiota in BBB formation (Braniste et al., 2014). During aging, dysbiosis of gut microbiota might lead to increased permeability of the gastrointestinal tract, which induces the increased level of circulating bacterial products, such as muramyl dipeptide (MDP) (Thevaranjan et al., 2017). In AD patients, the microbiota composition was characterized by decreased proportion of anti‐inflammatory bacteria (*E*.*rectale*) and increased abundance of pro‐inflammatory bacteria (*Escherichia*/*Shigella*), which was associated with the increased blood level of cytokines, such as IL‐6, NLRP3, and IL‐1β (Cattaneo et al., 2017). These elevated pro‐inflammatory cytokines accompanied bacterial products in the blood circulation might induce the production of NADPH and decrease tight junction proteins expression in BBB (Rochfort et al., 2014).

Vascular diseases, such as atherosclerosis, arteriolosclerosis, and stroke, promote BBB breakdown and make old patients more vulnerable to cognitive impairment (Chang et al., 2017; Skrobot et al., 2017; Wardlaw et al., 2017). Injured blood vessels, atheromatous plaque, and vascular occlusion induce the release of pro‐inflammatory cytokines and enroll immune cells to the damaged sites. In atherosclerosis, injured arteries are infiltrated with monocytes which lead to an increase of IL‐6 and activate macrophages (Tibolla et al., 2010). TNF‐α produced in vascular diseases induces the production of reactive oxygen species (ROS) and IL‐8, which further promotes vascular inflammation and cell infiltration (Hanrui Zhang et al., 2009). IL‐1 promotes the CNS‐specific inflammation through binding to interleukin‐1 receptor 1(IL‐1R1), a surface receptor on BBB endothelial cells. Activated IL‐1 signaling increases adhesion molecules expression, such as Vcam‐1 and Icam‐1 (Hauptmann et al., 2020). If the stimulated factors exist persistently, accumulated inflammatory factors release and sterile inflammation existence could damage the BBB integrity and increase its permeability, which facilitates the infiltration of pathogens and inflammatory cytokines in the brain and leads to neuroinflammation (Raj et al., 2015).

Intrinsic factors from CNS also induce the development of neuroinflammation, such as infection, death of neurons, Aβ peptide, and tau protein aggregation. Microglia take responsible for immune‐privileged and surveille the brain microenvironment, which could respond to the accumulated waste (Dukhinova et al., 2018). Besides, they also involve in regulating neuronal function and memory through mediating synaptic plasticity (Chu et al., 2019; C. Wang et al., 2020). The function of microglia is associated with its morphology (resting, hyperramified, amoeboid, and round) (Solano Fonseca et al., 2016). In the resting state, microglia release neurotransmitters, remodel synapses and perform as a surveillant to clear debris (Dá Mesquita et al., 2016). While in elderly human brains, microglia are activated by accumulated Aβ and tau protein in cortical tissues and are closely associated with cognitive decline (Felsky et al., 2019). In this condition, activated microglia show not only hyperramified and amoeboid phenotype but a shift to M1 phenotype (Chen et al., 2016). In the rodent model, though the composition of microglia clusters does not change during aging, special subpopulations of microglia are marked with inflammatory signals, such as Lgals3, cystatin F, and Ccl3, were significantly increased in aged brains (Hammond et al., 2019). Consistent with upregulated inflammatory markers, elevated inflammatory cytokines, and chemokines secreted by microglia are found in aged brain. In aged mice, IL‐6 mRNA expression level and supernatant IL‐6 from microglia are evidently increased compared with adult mice (Ye & Johnson, 1999). Increased IL‐6 binds to overexpressed IL‐6 receptor on microglia from aged mice and activates IL‐6 trans‐signaling pathway (Burton et al., 2013). Accordingly, microglia in such inflammatory state exacerbate neuron damage in aged brain (Hanisch & Kettenmann, 2007). Therefore, though microglia serve as “coordinator” between extracerebral and intracerebral environments, they will lose the role of protection with increased age and turn into an assistant of crimes.

## 4 MICROBIOTA, MICROGLIA, AND COGNITIVE DECLINE IN THE AGED

**FIGURE 2:**
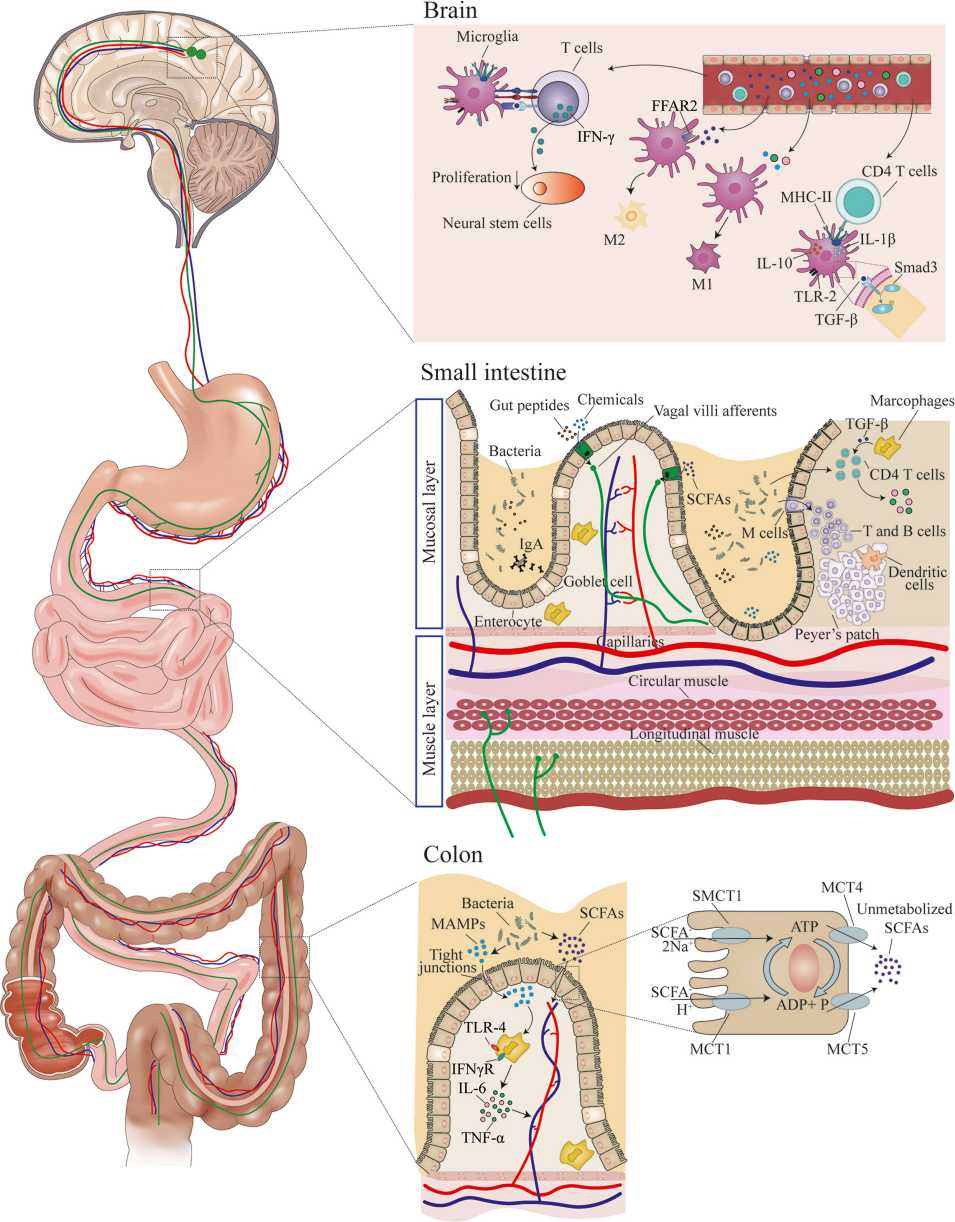
Microbiota, gut, and microglia connections. The communication routes between microbiota and microglia are depended on the location. In colon, MAMPs derived from gut bacteria approach to macrophages through disrupted tight junctions of intestinal tract barrier to stimulate IL‐6 and TNF‐α secretion, and SCFAs enter into capillaries by positive or passive transportation with the help of SMCT or MCT. In small intestine, metabolites from microbiota, such as gut peptides, chemicals, and SCFAs, could be absorbed by vagal villi afferents or leak into lamina propria through increased paracellular permeability to interact with immune cells (T cells, B cells, and macrophages), which might lead to the release of pro‐inflammatory cytokines including TGF‐β, IL‐6, and TNF‐α. Signals produced in the GI tract could enter into the bloodstream and activate vagus nerve or immune cells to communicate with microglia in the brain. Infiltrated CD4 T cells in the brain interact with microglia and induce upregulated expression of MHC‐II, IL‐10, IL‐1β, and TLR‐2 on the surface of microglia. TGF‐β binds to receptors on microglia and downregulates Smad3 signaling pathway. Interactions between microglia and T cells could also inhibit the proliferation of neural stem cells by IFN‐γ secretion. Pro‐inflammatory cytokines (IL‐6 or TNF‐α) from circulation mediate the state of the microglia and induce neuroinflammation in the aged brain

As microglia play a vital role in neurodegenerative diseases, abundant studies attempt to demonstrate the mechanism involved in it. In recent decades, scientists gradually realized that microglia and microbiota have amazing interplay. Microbiota influences the development, maturation, and function of microglia (Abdel‐Haq et al., 2019). In the aged, microbiota dysbiosis disturbs the microenvironment homeostasis and alters the state and function of microglia (Figure 2).

### 4.1 Microbiota, vagus nerve, and microglia

The vagus nerve connects the gut and brain through thousands of sensory and motor fibers. Accumulating evidence indicates that connections between gut microbiota and the vagus nerve appear to regulate the state of microglia and the level of inflammation in the CNS (Dinan & Cryan, 2017). To understand the communication between the specific bacteria in gut and the brain through the vagus nerve, we need to know how the bacteria can suppress or stimulate the signals transmitted through the vagus nerve. In the small and large intestine, vagal afferents terminate in the muscle layer as well as in the mucosal layer. In the muscle layer, vagal afferents end in longitudinal muscle and circular muscle to detect stretch and tension, thereby regulating intestinal movements. In the mucosal layer, vagal afferents are classified into two groups, vagal villi and vagal crypt afferents. Due to the close distance to the epithelial cell layer, gut peptides, metabolites, and chemicals absorbed by epithelial cells directly interact with vagus nerve endings.

A previous study showed that SCFAs secreted by gut bacteria, such as butyric acid, can activate vagal afferent in rat jejunum by a direct effect on afferent endings, while long‐chain fatty acids activate vagal afferents via a cholecystokinin‐dependent pathway (Lal et al., 2001). Consistent with this study, the release of bacterial byproducts induced by high‐fat diet leads to the response of vagal afferents terminals (Patterson et al., 2002). Besides, extracellular vehicles secreted by *Paenalcaligenes hominis* in GI of young mice could induce cognitive impairment, which can be inhibited by vagotomy (K.‐E. Lee et al., 2020). Similarly, oral administration of *Lactobacillus rhamnosus* induces a depression‐related behavior in adult mice through decreasing GABA receptor expression in the brain. This could also be attenuated by ablation of vagus nerve (Bravo et al., 2011; J. Zhang et al., 2020). Therefore, the gut microbiota might regulate signals of the vagus nerve by its metabolites.

Since changes caused by gut bacteria could suppress or stimulate vagal nerve terminals located in GI, signals transmitted by vagus nerve from GI to brain might influence the function of microglia (J. Kim et al., 2020). It is reported that non‐invasive vagus nerve stimulation causes a significant change of microglia branches number in mouse model of AD (Kaczmarczyk et al., 2018). If vagus nerve is damaged by vagotomy, increased microglia activation in hippocampus is observed in adult rats (Ronchi et al., 2012). Sen et al. revealed that obesity‐induced gut microbiota dysbiosis, especially an increase in *Clostridia* and *Bacilli*, downregulated IB4 binding and β3‐tubulin protein expression and therefore altered the vagal afferents from the gut and activated microglia (Sen et al., 2017). One possible pathway that contributed to microglia activation is acetylcholine (Ach) or norepinephrine (NE) pathway. In vitro incubation with Ach decreases IL‐1β and IL‐6 secretion and inhibits activation of JAK2/STAT3 and PI3K/AKT pathway in LPS‐induced microglia, therefore, switching microglia from M1 phenotype to M2 phenotype (Q. Zhang et al., 2017). Besides, activation of α7 nicotinic Ach receptor on microglia significantly prevents the release of inflammatory cytokines (De Simone et al., 2005). More studies are required to demonstrate the mechanisms that microbiota activates or suppresses microglia through which vagal afferents.

### 4.2 Microbiota, blood circulation, and microglia

Microbial metabolites, such as SCFAs and dopamine, are key contributions that gut microbiota modulates host metabolic reactions and health (Macfarlane & Macfarlane, 2003; Rekdal et al., 2019). These metabolites are secreted into intestinal tract, absorbed across the gut, and circulated in the host circulation (Figure 2).

SCFAs, known as saturated fatty acids with less than six carbon atoms, are mainly derived from gut microbial fermentation of dietary fiber (Miller & Wolin, 1996). Currently, at least 8 kinds of SCFAs have been found in human gut. Among them, acetate, propionate, and butyrate are the predominant ingredients of SCFAs, which accounts for over 95% of total SCFAs (Cook & Sellin, 1998; Wong et al., 2006). Once produced in the colon, SCFAs are transported across the apical and basolateral membranes of colonocytes. For the apical membrane, SCFAs are mainly transported by monocarboxylate transporters (MCTs). MCT1 transports SCFAs in an H^+^ ‐dependent electroneutral manner, while sodium‐dependent monocarboxylate transporter (SMCT) 1 transports SCFA anion coupled with Na^+^ in a ratio of 1:2 or 1:3 (Al‐Mosauwi et al., 2016; Coady et al., 2004, 2007).

After crossing apical membrane of colonocytes, SCFAs could enter into the citric acid cycle to generate energy for colonocytes (Tan et al., 2014). SCFAs that are not consumed in the colonocytes could be transported across the basolateral membrane and enter into portal blood. MCT4 and MCT5 are the main transporters localized to the basolateral membranes, which are responsible for the transportation of SCFAs (Gill et al., 2005). Though SCFAs could be transported into the bloodstream, only a small amount of acetate (36%), propionate (9%), and butyrate (2%) could measure in the blood circulation (Boets et al., 2015).

When arriving at the BBB, SCFAs mediate the functions of microglia through direct or indirect contact so that they can influence cognition of the host. Accumulating studies have indicated the evidence of SCFAs crossing the BBB. In rat model, intracarotid injection of ^14^C labeled butyrate and pyruvate leads to increased ^14^C‐SCFAs concentration in the brain (Sarna et al., 1979). In human, 22 nmol/g of propionate and 15 nmol/g of butyrate are measured in brain by using gas chromatography (Bachmann et al., 1979). These part of SCFAs, that go through the BBB, directly regulates the maturation and activity of microglia in the brain. In germ‐free mice, SCFAs administration decreases the CSFR1 expression on immature microglia surface and promotes the maturation of microglia by binding to SCFA receptor‐free fatty acid receptor 2 (FFAR2) (Erny et al., 2015). Butyrate, a member of SCFAs, is shown to reduce LPS‐induced inflammation not only in rats but also in mice models (Huuskonen et al., 2004; Yamawaki et al., 2018). Manually increased uptake of butyrate from dietary attenuates the release of pro‐inflammatory cytokines from microglia in aged mice (Matt et al., 2018), which might be regulated by nuclear factor‐kappa B (NF‐kB) and extracellular signal‐regulated kinase (ERK) signaling pathways (Park et al., 2005). Similarly, administration of *Clostridium butyricum* in APP/PS1 mice attenuates microglial activation, reduces TNF‐α and IL‐1β production, and improves cognitive impairment by suppressing the phosphorylation of NF‐kB p65 in microglia (J. Sun et al., 2020). Consistent with this, additional supplement of propionate by prebiotics reverses age‐related percentage of activated microglia in mice (Boehme et al., 2018). Aside from SCFAs that going across the BBB, SCFAs could also interact with BBB and might indirectly regulate the activation of microglia in the brain. In germ‐free adult mice, administration of *Clostridium tyrobutyricum* or *Bacteroides thetaiotaomicron*, which mainly produce SCFAs, decreases the permeability of BBB and increases the expression of occludin. Oral gavage of butyrate in germ‐free adult mice for 3 days receives the same effect on BBB (Braniste et al., 2014). In aged mice, application of *Lactobacillus* and sodium butyrate reverses cognitive impairment after operation by increasing the expression of tight junction proteins in BBB (Wen et al., 2020). During aging, increased permeability of BBB allows circulating pro‐inflammatory cytokines and pathogens (MDP and LPS) penetration (Thevaranjan et al., 2017; Zhan et al., 2018). SCFAs induced BBB tight junctions repair might prevent the microglia activation by contacting with circulating inflammatory cytokines. However, there is still a lack of direct evidence to illustrate this axis. More studies are required to demonstrate the mechanism involved in SCFAs mediated BBB permeability regulation.

### 4.3 Microbiota, immune cells, and microglia

Communications between microbiota and immune cells in the host have been recognized for decades. Growing evidence indicates that gut microbiota might interact with the peripheral immune system to indirectly modulate the function of microglia in the brain (Figure 2).

In the periphery, microbiota‐originated molecular patterns, including lipopolysaccharide (LPS), bacterial flagellum, and CpG DNA, are also known as microbial‐associated molecular patterns (MAMPs), which could mediate the functions of immune cells including macrophages, dendritic cells, and neutrophils (Timothy R Sampson & Mazmanian, 2015). Age‐related gut microbiota dysbiosis increases the paracellular permeability of colons and allows LPS to leak into blood circulation. This induces TNF and IL‐6 release and deteriorates the ability of bone marrow‐derived macrophages (BMDMs) to extinguish *Streptococcus pneumoniae* (Thevaranjan et al., 2017). Additionally, deficiency of pro‐inflammatory cytokines secretion in macrophages lowers the ability of macrophages to support B cells response to LPS in aged mice (Chelvarajan et al., 2006). Dysfunction of BMDMs in aged rats increased the responsiveness to LPS in vitro by increasing the expression of IFNγ receptor and toll‐like receptor 4 (TLR4) (Barrett et al., 2015). Inflammatory signals produced by dysfunctional peripheral macrophages in old age transduce to microglia not only via leaked BBB in old brain but also meningeal cells (Liu et al., 2013). After receiving these inflammatory signals, microglia displays a hyperactive response in aged rodents, which presents as upregulation of major histocompatibility complex class II (MHC‐II), IL‐1β, IL‐10, and TLR‐2 (Henry et al., 2009). Meanwhile, IL‐4‐induced M2 promoting effects are less sensitive in aged microglia (Fenn et al., 2012). Some signaling pathways in microglia are downregulated, such as transforming growth factor β (TGF‐β1), mothers against decapentaplegic homolog 3 (Smad3) pathway (Tichauer et al., 2014). These changes in microglia might lead to enhanced neuroinflammation and synapse remodeling in aged mice (Kondo et al., 2011).

T cells also involve in the gut microbiota‐induced microglia function regulation. Groups of specific T cells diffuse almost everywhere in the human body to surveille the peripheral environment, discriminate antigens, and extinguish pathogens. In the gut, T cells are not only accumulated in Peyer's patches to recognize the luminal antigens sampled by M cells in the follicle‐associated epithelium, but resided in the lamina propria as CD4^+^ T cells to mediate the immune response (MacDonald & Monteleone, 2005). During the aging process, the integrity of gut barrier is decreased as a result of passive factors, including increased epithelial apoptosis, overactivated c‐Jun N‐terminal kinase (JNK) signaling pathway in intestinal epithelial stem cells and decreased expression of tight junctions proteins (Branca et al., 2019; Farhadi et al., 2003). Besides, aging‐associated gut microbiota dysbiosis also contributes to increased intestinal permeability (Thevaranjan et al., 2017). In this situation, metabolites from gut microbiota, pro‐inflammatory cytokines, and even bacteria translocate into lamina propria through leaky gut epithelial barrier, which induces T cells to produce IL‐6 and TNF‐α (Heumann et al., 1994). Results from rhesus macaque models indicate that lower levels of *Firmicutes* and higher levels of *archaeal* and *proteobacterial* species in aged animals are associated with chronic systemic inflammation and increased memory CD4 T cells in the brain (Pallikuth et al., 2021).

However, in which way do activated T cells in the gut influence the functions of microglia in the brain? Inflammation induced by T cells in the gut could lead to chronic systemic inflammation as a result of immune homeostasis damage in aged individuals. Age‐driven changes in regulatory T cells (Tregs) weaken their ability to suppress immune reactions, such as IL‐17 and IL‐2 secretion by T cells, which deteriorates the imbalance of the immune homeostasis and promotes inflammation in the body (Churov et al., 2020). These reactive T cells activated in the peripheral immune system infiltrate in the brain partly as a result of gut antigens activation (Mora et al., 2003). Growing evidence indicates that inflammatory activities increased the number of dendritic cells and T cells in 12 months old mice (Stichel & Luebbert, 2007). Though the mechanism of T cells recruitment in the brain is not clear, single‐cell analysis in old brains reveals that T cells infiltrate in old brain neurogenic niches and express interferon‐γ, which inhibits the growth of neural stem cells (Dulken et al., 2019; Moreno‐Valladares et al., 2020). As microglia are the immune cells resided in the brain, infiltrated T cells might have a crosstalk with microglia to modulate the functions of the aged brain. Previously, isolated MHC‐II^+^ microglia are proved to effectively support the activation of CD4^+^ T cells, which is stimulated by an encephalitogenic myelin basic protein, to produce IFN‐γ and TNF‐α not IL‐2 (Ford et al., 1996). Similarly, under specific in vitro conditions, microglia expressing MHC‐II induces the activity of CD4 regulatory T cells by low‐dose IFN‐γ (Ebner et al., 2013). In AD mice model and human AD specimen, Iba‐1^+^ and CD68^+^ microglia are found to be colocalized with CD8^+^ T cells at the site of Aβ plaques, which indicates the interaction between microglia and T cells in the brain (M. S. Unger et al., 2018). C. Laurent et al. reveals that chemokine (C‐C motif) ligand 3 (CCL3) exclusively produced by microglia in hippocampus of AD mice model facilitates CD8^+^ T cells infiltration, and elimination of these infiltrated T cells restores the cognitive function of AD mice (Laurent et al., 2017). In this study, unfortunately, it is unknown whether microglia depletion could attenuate CD8^+^ T cells infiltration in the hippocampus. Unexpectedly, microglia ablation by PLX5622, a colony‐stimulating factor 1 receptor (CSF1R) inhibitor, increases the number of CD3^+^/ CD8^+^ T cells in the brain of APP/PS1 mice, while this treatment has no improvement on cognition of APP/PS1 mice (M. Unger et al., 2018). Increased CD8^+^ T cells in the brain might neutralize the influence of microglia ablation on neuroinflammation, which makes PLX5622 fail to restore the cognitive functions. Furthermore, microglia in aged brain partly switch to phagocytic phenotype as a response of accumulating myelin pathology, which also leads to cognitive impairment (Shobin et al., 2017). These data suggest that neuroinflammation induced by crosstalk between microglia and infiltrated T cells in the aged brain plays a vital role in cognitive deficits.

## 5 MICROBIAL DYSBIOSIS AND MICROGLIAL DYSFUNCTION IN NEURODEGENERATIVE DISEASES

Considering the wide range of physiological functions of microglia in brain development and homeostasis maintenance, microglia are believed to participate in the pathogenesis of several neurodegenerative diseases. As we discussed above, the morphology and functions of microglia are affected by factors and signals originated from brain or the periphery. Therefore, it has been recognized that several neurodegenerative diseases are not only originated in the brain but affected by periphery factors. Here, we briefly discuss the potential role of microbiota‐microglia connections in AD and PD.

### 5.1 AD

AD is characterized as a neurodegenerative disease with slowly but progressively loss of memory, cognition, and motor functions (Burns & Iliffe, 2009). Currently, the recognized pathological hallmarks of AD are amyloid plaques deposition and tau protein aggregation (Savelieff et al., 2013). Factors in the brain and peripheral environment involved in the pathogenesis of AD, eventually leading to severe neuroinflammation and neurons loss.

Microglia, as the resident phagocytes in the brain, play an important role in Aβ clearance contributing to homeostasis of Aβ production and removal (C. Y. D. Lee & Landreth, 2010). Continuous exposure to Aβ species, microglia switch to an activated state with increased proliferation and expression of CD36, iNOS, and CD14 (Martin et al., 2017; Olmos‐Alonso et al., 2016). Activated microglia, on the one hand, lead to Aβ clearance by increased phagocytosis. Pattern recognition receptors (PRPs) expressed on the surface of microglia drive microglia binding to different species of Aβ, and microglia rapidly transport these plaques into endolysosomal compartments to degradate (Mandrekar et al., 2009; C. Venegas & Heneka, 2017). On the other hand, chronic activation of microglia results in chronic neuroinflammation and neurotoxicity by pro‐inflammatory factors release. Long‐term binding of Aβ peptides to PRPs induces enhanced release of pro‐inflammatory cytokines in microglia, such as IL‐1β and TNF‐α, which could promote the progression of AD (Q. Li et al., 2018). Therefore, it seems that the function of microglia is dependent on the environment around microglia.

Considering the potential interplay between the gut microbiota and microglia, bacteria in the gastrointestinal tract might regulate the pathogenesis of AD. Changed composition of gut microbiota is observed several mice models of AD and AD patients. The decreased abundance of *Firmicutes*, *Verrucomicrobia*, *Proteobacteria*, and *Actinobacteria* is observed in old age of AD mice models (Harach et al., 2017). Additionally, abundant phyla proportion of *Firmicutes* and *Bacteroidetes* and families of *Lachnospiraceae*, *Ruminococcaceae*, and *Bacteroidaceae* are observed in AD patients (Vogt et al., 2017). Support for the hypothesis that microbiota‐microglia connections involve in AD development, and progression was that gut metabolites such as SCFAs could affect the development of AD by mediating the function of microglia and neuroinflammation (J. Sun et al., 2020). SCFAs level was found to be altered in AD patients compared with healthy controls (Nagpal et al., 2019). A recent study indicated that SCFAs supplementation in germ‐free AD mice increases microglia recruitment to Aβ plaques and promotes Aβ deposition likely by mediating the phenotype of microglia (Colombo et al., 2021). Previously, Venegas et al. suggested that microglia promote Aβ plaques deposition in AD mice model by secreting ASC specks, which are pro‐inflammatory protein complexes (Carmen Venegas et al., 2017). However, loss of Trem2 accelerates amyloid seeds due to reduced Aβ clearance, accompanied with reduced plaque‐associated ApoE (Parhizkar et al., 2019). Therefore, it seems that the function of microglia is largely depended on the peripheral environment.

### 5.2 PD

PD is defined as a long‐term degenerative disease occurred in the CNS that mainly affects the motor system. The common symptoms of PD are resting tremor, rigidity, and slowness of movement (Politis et al., 2010). And the main pathological changes in PD are cell death in basal ganglia which mainly leads to death of dopaminergic neurons (Davie, 2008). Complicated interactions between genetic and environmental factors are believed to cause the pathogenesis of PD (Kalia & Lang, 2015).

The evidence that microglia involve in the pathogenesis of PD is revealed by several studies. Sruti et al. found that p.R47H variant in Trem2 of microglia is a risk factor for PD (Rayaprolu et al., 2013). Besides, positron emission tomography (PET) revealed that the activation of microglia is associated with the early pathogenesis of PD possibly by releasing cytokines (Gerhard et al., 2006). The distribution of microglia is specific in PD, which predominantly distributed in substantia nigra and striatum (Grabert et al., 2016). This leads to region‐specific release of cytokines, such as TNF‐α and IL‐6, affecting the death of dopaminergic neurons (Nagatsu & Sawada, 2005). Microglia are also stimulated by α‐synuclein (a misfolded protein in Lewy bodies) and promote the release of pro‐inflammatory cytokines in turn (George et al., 2019). In summary, these results support that microglia play a pivotal role in progression of PD.

Though PD is a brain disorder mainly affected the function of the brain, recent evidence suggests that factors from gastrointestinal tract might play a role in pathogenesis of PD. The composition of gut microbiota in PD patients is significantly changed compared with healthy controls. Lin et al. found that the diversity of gut microbiota in PD patients is less than that of healthy controls (Lin et al., 2019). Increased abundance of *Verrucomicrobia*, *Prevotella*, *Mucispirillum*, *Porphyromonas*, *Lactobacillus*, and *Parabacteroides* was observed in PD patients, and dysbiosis of gut microbiota was associated with elevated inflammatory response (Lin et al., 2019). Meanwhile, metabolites derived from gut microbiota are altered in PD patients, which presents as markedly reduced SCFAs (M. M. Unger et al., 2016). It should be noticed that SCFA‐producing KEGG pathways are upregulated in mice who received fecal transplants from PD donors (T. R. Sampson et al., 2016). The indications that microbiota‐microglia connections might drive the pathogenesis of PD are based on the following observations. Germ‐free mice that overexpressed human α‐synuclein display motor dysfunction, GI dysfunction, and microglial activation (T. R. Sampson et al., 2016). Conducting oral gavage for these mice with mixture of SCFAs induces α‐synuclein aggregation and morphological changes of microglia, which promotes motor deficits (T. R. Sampson et al., 2016). Another study also observed reduced microbial dysbiosis, reduced SCFAs level and microglia activation, and alleviated motor deficits in PD mice when received fecal transplants from healthy mice (M.‐F. Sun et al., 2018). Though these findings reveal part role of microbiota‐microglia connections in PD, the precise mechanism that gut microbiota affects motor deficits and neuroinflammation is still unclear.

## 6 FUTURE PERSPECTIVES

Aging is a major risk of neurodegenerative diseases, such as AD. In recent years, microglia, the resident immune cells in the CNS, are revealed to involve in the development of neuroinflammation and be mediated by gut microbiota due to the involvement of microbiota in microglia maturation and function. To date, however, the studies likely only give a glimpse into the tip of the iceberg that the interplay between the gut microbiota and microglia during aging. The emergence of new tools, such as single‐cell analysis and spatial transcriptomic sequencing (ST‐seq) used to feature the microbiota and microglia, will accelerate the further recognition on the crosstalk between gut microbiota and microglia (Masuda et al., 2020). Recently, a multifunctional technology, named high‐phylogenetic‐resolution microbiota mapping by fluorescence in situ hybridization (HiPR‐FISH), achieves identification of taxa in situ only with a single round of confocal imaging and reveals the spatial network disruption of gut microbiota in mice treated with antibiotics (Shi et al., 2020). Apart from in vitro studies, noninvasively in vivo imaging and tracking of gut microbiota could greatly promote the knowledge in microbiota‐microglia interactions. Previously, metabolic oligosaccharide engineering and biorthogonal click chemistry were used to label commensal bacteria in the gut and demonstrated the distribution and colonization of labeled bacteria in vivo by two‐photon microscopy and non‐invasive whole‐body imaging (Geva‐Zatorsky et al., 2015). Radiolabeled probes (^18^F‐FDS and ^64^Cu‐NOTA‐DBCO) could also achieve in vivo imaging of gut microbiota by using PET/CT (Y. Wang et al., 2020; Z. Zhang et al., 2021). Meanwhile, the developed microscopy techniques, such as two‐photon microscopy, multiphoton microscopy and allows to observe the activity of microglia in the living brain (Hierro‐Bujalance et al., 2018; Zhao et al., 2018). These studies shed light on the characterization of comprehensive changes in gut microbiota and microglia of aged individuals, promoting the screening of specific markers in cognitive aging and speeding the development of microbiota or microglia‐targeted therapies.

One of the priority questions remains to be clarified is what the certain mechanism involved in the phenotypic and functional changes of microglia by altered gut microbiota. It seems that gut microbiota changes and microglia alterations may interact as both cause and effect. The observed changes of gut microbiota might be a cause or result of neuroinflammation or microglia alterations. Metabolites or neurotransmitters derived from gut commensal microbiota have the potential to attenuate and even restore the age‐induced alterations of microglia and finally improve cognitive impairment. Therefore, further investigations are called to reveal the amazing interplay between gut microbiota and microglia so that the microbiota or microglia‐targeted therapies come true and benefit the patients. Additionally, technologies development in noninvasively and in vivo monitoring the composition of gut microbiota and functions of microglia will greatly accelerate the clinical practice of these therapeutic strategies.

## CONFLICT OF INTEREST

The authors declare no conflict of interest.

## AUTHOR CONTRIBUTIONS

MT, LC, HZ, and WC conceived the manuscript. RZ finished the manuscript and figures. SQ summarized the tables. JZ contributed to the manuscript revision and Figure 2 preparation. All authors contributed to the manuscript revision.
